# Influence of Pre- and Postharvest Summer Pruning on the Growth, Yield, Fruit Quality, and Carbohydrate Content of Early Season Peach Cultivars

**DOI:** 10.1155/2014/104865

**Published:** 2014-03-10

**Authors:** Ali Ikinci

**Affiliations:** Department of Horticulture, Faculty of Agriculture, Harran University, 63330 Sanliurfa, Turkey

## Abstract

Winter and summer pruning are widely applied processes in all fruit trees, including in peach orchard management. This study was conducted to determine the effects of summer prunings (SP), as compared to winter pruning (WP), on shoot length, shoot diameter, trunk cross sectional area (TCSA) increment, fruit yield, fruit quality, and carbohydrate content of two early ripening peach cultivars (“Early Red” and “Maycrest”) of six years of age, grown in semiarid climate conditions, in 2008 to 2010. The trees were grafted on GF 677 rootstocks, trained with a central leader system, and spaced 5 × 5 m apart. The SP carried out after harvesting in July and August decreased the shoot length significantly; however, it increased its diameter. Compared to 2009, this effect was more marked in year 2010. In general, control and winter pruned trees of both cultivars had the highest TCSA increment and yield efficiency. The SP increased the average fruit weight and soluble solids contents (SSC) more than both control and WP. The titratable acidity showed no consistent response to pruning time. The carbohydrate accumulation in shoot was higher in WP and in control than in SP trees. SP significantly affected carbohydrate accumulation; postharvest pruning showed higher carbohydrate content than preharvest pruning.

## 1. Introduction

Small, dwarf, or size controlled fruit trees provide easier pruning, thinning, spraying, and harvesting and could lead to production of high-grade fruit at lower production cost [1]. To induce dwarfing, fruit growers can use dwarfing rootstocks. However, dwarfing rootstocks are not yet available for peaches, like apple [2].

Summer pruning has long been used as a management method for fruit trees. It was shown to be a valuable method of controlling tree growth [3–7], increasing flowering [4], increasing fruit color [1, 3, 7, 8], increasing soluble solids concentration (SSC) [5–7, 9], increasing flower bud formation [10], and decreasing titratable acid content (TA) [1, 6, 7]. Disadvantage of summer pruning include reduced cold hardiness of flower buds [11], delayed defoliation [7, 11], carbohydrate levels in the tree [11–13], fruit size [2, 8, 14, 15], and trunk enlargement [14].

The above mentioned differences can be attributed to differences in timing and severity of pruning, and because, in some cases, summer pruning was used as a replacement for dormant pruning rather than as a supplement.

Summer pruning of peach trees, at a time when stems, fruits, and roots are still growing, potentially could remove 35% to 45% of the total tree leaf area. The significant loss of leaf area on summer-pruned trees may lead to a reduction in the carbohydrate and nutrient element concentrations in remaining tissues and thus limit the growth of trees. Summer pruning decreased carbohydrate concentration in stems and roots of mulberry and reduced the leaf carbohydrate concentrations by about 30% during the 45-day period after pruning [16]. In sweet cherry, after summer pruning, the middle and upper parts of the trunk contained the highest concentrations of starch and soluble sugars. One year after summer pruning, the level of carbohydrates in the trunk was lower compared with unpruned trees [12]. Total carbohydrate contents of perennial parts of trees in the temperate zone reach a maximum in the autumn, begin to decrease in late winter, and decrease rapidly in early spring [12, 17].

Previous studies have shown that pruning results in quantitative changes in carbohydrate reserves. Pruning affects the level of reserves by elimination of storage sites [18]. Pruning has been also reported to slow down the reconstitution of reserves or to contribute to their depletion [19].

Carbohydrates are an essential source of reserve energy in temperate zone trees and other perennial plants. They can be mobilised for metabolism or translocated to other plant organs. The concentration and localization of carbohydrates, such as sugars and starches, within tissues are affected by many factors, such as temperature, moisture, light, pruning, and time of planting [20].

The aim of this study was to determine the effect of preharvest (May and June) and postharvest (July and August) summer pruning on accumulation of carbohydrates in shoots, vegetative and reproductive growth, fruit quality, and yield efficiency of the two early ripening peach cultivars “Early Red” and “Maycrest” on GF 677 rootstock.

## 2. Materials and Methods

This research was conducted during the 2008–2010 growing seasons at the Harran University (Şanlıurfa, Turkey) Pome Fruit Research Station (37°19′N; 38°96′E; 520 m a.s.l.). Şanlıurfa province has continental climatic features; it is very cold and wet in the winter and very hot and dry in the summer. During the experiment, the air temperatures were in average of 29.6°C in summer and 6.4°C in winter, while annual precipitation ranged between 428 and 486 mm, mainly concentrated between the months of November and April. The soil in the orchard (0–40 cm) is loamy with 40% clay, 33.2% silt, and 21.4% sand, low in organic matter (1.1%), and rich in calcium carbonate contents (25%) and has a high pH (8.4) [21].

### 2.1. Plant Materials, Treatment, and Measurement

Summer pruning was performed on six-year-old early ripening trees of “Early Red” and “Maycrest” peach on GF 677 rootstock. Trees were planted in 2004 at a 5 × 5 m (400 trees ha^−1^) spacing and trained to a central leader system, drip irrigated, and managed using standard cultural practices.

Trees were selected for uniformity based on tree size, trunk circumference, and total fruit count. Trees were *≈*2.5 to 3.0 m high and 2.5 to 3.5 m wide. All trees had been uniformly pruned during previous dormant season. Dormant pruning was performed in February and consisted of heading vertical shoots to maintain tree height 2.5 m, thinning cuts, and removal of vigorous watersprouts. During 2008 to 2010, the following treatments were applied to trees: (a) unpruned control; (b) pruned in May 7; (c) pruned June 7; (d) pruned July 7; and (e) pruned August 7.

All summer-pruned trees received normal winter hand pruning during February 2009 and February 2010. These trees are named the SP and WP, respectively. Summer pruning consisted of heading back of current season shoots to about 10 cm length and removal of vigorous shoots [7, 13, 22]. *≈*50% of the current season's shoot growth was removed by each of summer pruning treatments. In addition, diseased and broken branches were also removed from trees [1, 7, 11, 23]. Control trees received only light dormant (winter) pruning with thinning-out cuts. The fresh weight of prunings (kg tree^−1^) was determined for all treatments at each pruning date. In 2008, all SP treatments on the trees and presummer pruning (SP) have been considered as applications and all data obtained are not included in the calculations. Treatments were arranged in a randomized complete block design with four single-tree replications per treatment.

Ten shoots at 1.5 m above ground were selected from around each tree for growth measurements. The terminal and lateral shoots were measured prior to summer pruning treatments and in November. Shoot diameter was measured at 2 cm from the shoot base. For calculating the trunk cross-sectional area (TCSA), trunk circumference about 20 cm above the graft union was measured with a hand caliper at the end of the growing season and converted to TCSA in cm^2^. Yields per tree were recorded in years 2009 and 2010. Finally, yield efficiency was measured as yield per tree divided to TCSA in late growing season (yield per tree/TCSA). The TCSA and shoot growth were determined by Marini [11].

Ten fruits per tree were randomly selected and used to determine mean fruit weight, total soluble solids content (SSC), and titratable acidity (TA). The SSC (%) was measured with a hand Atago refractometer. TA of fruit juice was measured by titrating fruit juice against 0.1 N NaOH at pH 8.1 and was expressed as percent malic acid.

### 2.2. Carbohydrates in the Bark of Dormant Shoots

After summer pruning in 2009 and 2010, a random sample of 12 annual shoots was collected (at the beginning of rest period/in December) from each tree. Phloem with cambium was used and prepared for analyses. Barks with a knife peeled branches were dried at 70°C for at least 72 hr and then frozen at −18°C, lyophilized, and stored in a desiccators at −18°C for carbohydrate analysis. The reduced sugar, total sugar, and starch contents were determined by dinitrophenol and anthron methods [24].

### 2.3. Statistical Analyses

Data were evaluated by analysis of variance with Minitab 16.1.0 Statistics software package. When the *F*-test was significant, means were separated by Duncan's multiple range test (DMRT) at *P* < 0.05. An arcsine square-root transformation was performed on percent data.

## 3. Results

### 3.1. Average Fresh Weight of Prunings (kg tree^−1^)

**Figure 1:**
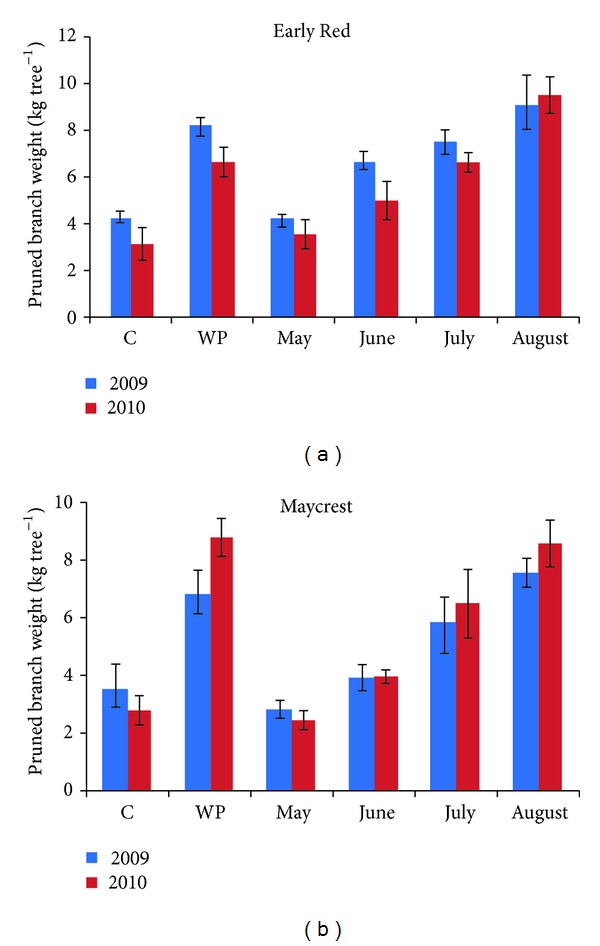
Effect of summer and winter pruning on pruned shoot weight of peach trees in 2009-2010. Vertical bars represent SE (*n* = 6).

Generally, more fresh weight was removed as the pruning date was delayed from May to August (Figure 1). The least prunings were found in the control trees in 2009 and 2010 in Early Red cultivar. Whereas, summer pruning done in May of both years in Maycrest peach cultivar is the practice from which the least prunings were obtained.

### 3.2. Average Shoot Length and Diameter

Pruning treatments had significant effect on the enlargement of shoot length and shoot diameter in both peach varieties (Table 1). The longest shoot enlargement of Early Red and Maycrest peach was detected in trees with control and WP treatment in 2009 and 2010. It was found that the length of the shoot obtained after the SP conducted in May on both peach varieties was longer compared to other SP treatments. In the second year, it was found that the shoot growing significantly decreased in WP and SP treatments compared to the first year, except for control trees. It was also noticed that postharvest SP conducted on peach trees had a significant effect on reducing the rate of shoot enlargement of trees compared to preharvest SP.

The highest shoot diameter enlargement was detected in trees with control and WP treatment in both peach varieties, similar to shoot length enlargement. In the second year of SP treatments, shoot diameter enlargement of Early Read peach trees increased, compared to the first year. However, this increase was observed in trees with SP treatment in May, June, and July for Maycrest trees. In general, it was found that SP treatments in 2010 led to a significant increase in shoot diameter compared to the first year.

### 3.3. Trunk Cross Sectional Area (TCSA)

The effects of pruning on TCSA (cm^2^), yield (kg tree^−1^), and yield efficiency (kg cm^−2^ TCSA) of Early Read and Maycrest peach cultivars are presented in Table 2. The effects of pruning treatments were found to be significant on TCSA development of peach trees in both of the years and the effect of pruning by varieties and years varied. In Early Read cultivar, the highest TCSA enlargement was obtained from SP-August and control in 2009 and from control and SP-May treatments in 2010. In Maycrest variety, the highest TCSA enlargement was obtained from trees with WP treatments in both of the years. In Early Read, the lowest TCSA enlargement was obtained from SP-June and WP in 2009 and from SP-June treatments in 2010 and in Maycrest, the lowest TCSA enlargement was obtained from SP-July treatments in both of the years.

### 3.4. Yield and Yield Efficiency

Pruning treatments had statistically significant effects on the fruit production of peach trees (Table 2). In Early Read and Maycrest varieties, the highest fruit production was obtained from control trees in both of the years. In Maycrest variety, following control trees, the highest production was obtained from WP treatment. In Early Read, the effects of pruning treatments on fruit production differed by treatment period.

There were statistically significant differences (*P* < 0.05) among applications on yield as determined by TCSA (kg·cm^−2^) (Table 2). Similar to fructification values, the highest yield efficiency value of both peach trees was obtained from control trees which were not pruned. It was observed that yield efficiency values of trees with SP treatments were lower than those of trees with control and WP treatment and SP-May had low effect on production value. On the other hand, it was found that yield efficiency value of both preach varieties in 2010 was lower compared to the year 2009.

### 3.5. Average Fruit Weight

Early Read and Maycrest varieties were harvested in 26 June and 07 June in 2009 and 21 June and 1 June in 2010, respectively. Pruning treatments had significant effect on average fruit weight of peach trees varieties (Table 3). In Early Read, the heaviest fruits were obtained from SP-May treatment in 2009 and 2010 (117.46 g and 122.87 g, resp.). In Maycrest, the heaviest fruits were obtained from SP-May treatment in 2009 (121.90 g) and from SP-June treatment in 2010 (124.75 g). In general, the smallest fruits were obtained from control trees in both peach varieties.

### 3.6. Soluble Solids

Pruning treatments did not affect the SSC of Early Read peach fruits in 2009 and 2010, whereas SSC in Maycrest was affected by pruning treatment only in 2009 (Table 3). Data from this study confirm that SP or WP had inconsistent effects on peach fruit SSC. As can be seen in Table 3, in the second year of pruning treatments, an increase was observed in WP and some SP treatments of the SSC compared to the first year.

### 3.7. Titratable Acidity

Pruning treatments conducted in different periods had significant effects on TA (%) of fruits of Maycrest variety in 2009 and 2010 and of Early Read in 2010 (Table 3). The TA of peach varieties differed by pruning period and years to a great extent. The TA analyses results, as seen in Table 3, was lower in 2010 compared to the year 2009.

### 3.8. Carbohydrate Concentrations

Pruning had a significant effect on starch and total carbohydrate contents of two peach cultivars (Table 4). The effects of SP or WP on carbohydrate concentrations of peach trees have been quite variable.

### 3.9. The Effect of Pruning on Starch Content (%)

Pruning applications significantly affected starch contents of peach shoots (Table 4). In Early Read variety, the highest starch level was found in trees with control (5.50%) and WP treatment (5.11%) in 2009 and in control trees (5.70%) in 2010. In Maycrest variety, the highest starch value was obtained from trees with WP treatment (5.50% and 7.30%, resp.) in 2009 and 2010. In Early Read variety, the lowest starch value was obtained from SP-June (2.50%) treatment in both of the years. In Maycrest variety, the lowest value was obtained from SP-May (3.22%) in 2009 and from SP-July (4.95%) in 2010.

### 3.10. The Effect of Pruning on Total Extracted Carbohydrate Concentrations

As for total carbohydrate concentrations (CHO) calculated by adding starch concentrations and reducing sugars, it was found that pruning treatments had significant effect on peach trees (Table 4). Although there were variables by years in both peach varieties, the highest CHO level was obtained from trees in control and WP treatment. In general, it was found that SP treatments decrease CHO level in peach shoots and this decrease was in higher amounts in SP-June treatments in Early Read and in SP-May treatments in Maycrest.

### 3.11. The Effect of Pruning on Total Extracted Carbohydrate Concentrations

As for total carbohydrate concentrations (CHO) calculated by adding starch concentrations and reducing sugars, it was found that pruning treatments had significant effect on peach trees (Table 4). Although there were variables by years in both peach varieties, the highest CHO level was obtained from trees with control and WP treatment. In general, it was found that SP treatments decrease CHO level in peach shoots and this decrease was in higher amounts in SP-June treatments in Early Read variety and in SP-May treatments in Maycrest variety.

## 4. Discussion

In the research, all application trees to be pruned in summer were pruned in 2008 at the same date; therefore, prunings values in 2009 were more realistic. Branch weight is much more since the branches, which are removed in summer pruning, include leaves. Taylor and Ferree [25] found that summer pruning reduced whole tree dry weight in proportion to the percentage of shoot removal. If the branch weight obtained from pruning was determined in terms of dry weight in the research, branch weight obtained from winter pruning would be weightier by 2- to 3-fold than the summer pruning from which the prunings at the highest number were obtained. Summer pruning in 2009 in peach trees led to proportional, not numeral, reduction of both summer and winter prunings weights in 2010. The results obtained from the study in terms of prunings' weights received from pruning practices are similar to the results of Miller [10] obtained from apple; Kappel and Bouthillier [26] obtained from peach; İkinci [7] obtained from almond, peach, and apricot; and Hossain et al. [6] obtained from peach. Kappel and Bouthillier [26] stated that fewer prunings were obtained in winter pruning from trees whose summer pruning was done. They stated also that the weight of shoots obtained in summer pruning in the second year was less than the first year.

Pruning treatments conducted on two peach varieties (WP and SP), especially the SP decrease shoot growth significantly. Restricted shoot growth and reduced shoot diameter started to be observed in the second year (Table 1). It was found that postharvest or late July-August SP was more effective on slowing down the shoot growth compared to preharvest or early May-SP and June-SP. Studies on peach and apple trees support our research results [1, 6, 7, 9, 14, 27–29].

Increase trunk enlargement of peach trees with control and WP was higher compared to SP. Many other researchers studying on pruning of peach [7, 14, 28, 30] and other tropical fruit varieties reported that SP decreases trunk enlargement compared to WP. However, studies on apple [25, 31] suggested that SP has no effect on trunk enlargement.

Although variable results were obtained from SP and WP treatments conducted by years and varieties, the yield efficiency value of trees with control and WP treatments in the first year was higher. Likewise, Daulta et al. [32], Miller [22], Tehrani and Leuty [8], Chitkara et al. [33], Küden and Kaşka [23], İkinci [7], Akçay [34], and Demirtaş et al. [5] all found different results on the effect of pruning on yield and yield efficiency data in different temperate fruit trees. We found that trees with SP had close values to control trees or to those with only WP in terms of yield efficiency values in second year pruning treatments (Table 2).

It was reported in previous pruning studies that summer pruning on apple, almond, peach, and apricot decreases yield efficiency compared to winter pruning. Demirtaş et al. [5] reported that preharvest and postharvest period pruning on “Hacıhaliloğlu” apricot variety improve the yield of trees; yet this increase is not statistically significant. Similarly, Bayazit et al. [3] reported that there is no statistically significant difference between summer-pruned and unpruned trees of peach and some nectarine varieties in terms of yield per tree.

Fruit set of peach trees occurs on one, two, or multiyear branches. The application of WP + SP treatment on peach trees has thinning effect on fruits of trees in the same year. Our findings indicate that if SP treatment is performed on the same trees after WP in preharvest period (e.g., 30 days), nonharvested fruits on the trees grow larger compared to trees on which WP treatment is not applied.

Various researchers studying on pruning explained that SP leads to an increase on the size of fruits as follows. The fruit size distribution effects of SP may be the result of a decrease in total leaf area and, as a result, a decrease in total transpirational loss by tree. Such trees would use less water and be less susceptible to water stress, thereby improving fruit water status and fruit growth rate during Stage III (final swell) when the fruit have a large demand for photosynthates and water [13, 35–37]. There may have been an increase in photosynthate available to fruit of summer-pruned trees due to an increase in photosynthetic photon flux density and/or the removal of competitive sinks, that is, watersprouts. Also, improved light exposure may have strengthened fruit sink activity, thus increasing fruit size [4].

Mika [38] reported that SP does not increase the largeness of fruits; on the contrary, he asserted that formation of smaller fruits results from the 20% decrease in assimilate amount, due to the reduction of leaves that provide resource for assimilate pool of trees by pruning. Greene and Lord [31] reported that SP reduced apple fruit weight in 1978–1980 and increased it in 1979.

Gür and Pırlak [39] reported that average fruit weight differs between 133.4 g and 258 g in 16 peach varieties grafted on peach seedling in Eğirdir conditions in western Turkey. Bayazit et al. [3] reported that average fruit weights of 5 different peach varieties with SP differ between 64.59 g (Spring Belle) and 95.79 g (Springcrest). The average fruit weights (minimum: 78.74 g, maximum: 124.75 g) obtained in the present study which was conducted in Şanlıurfa province under semiarid climate conditions are less than the values in the research conducted by Gür and Pırlak [39] and relatively higher than the results of Bayazit et al. [3] (Table 3).

In pruning treatments conducted on early peach varieties successively in 3 years, SSC values of 2010 were higher than those of 2009. According to earlier studies, Daulta et al. [32], Hossain et al. [40], and Hossain and Mizutani [1] reported that SP applications had increased SSC in peach. However, Niran [15], Miller [10], İkinci [7], and Bayazit et al. [3] reported that SP applications had no significant effect on SSC of fruits in both peach and apple. Marini and Barden [41], Taylor and Ferree [25], Cust and Ferree [42], Miller [22], and Christopher et al. [37] stated that SP applications negatively affected SSC in peach. The reduction in fruit size and fruit soluble solids associated with relatively severe SP is likely due to the reduction in total photosynthetic production of tree resulting in less carbohydrates available for the fruit.

As a result of the effect of pruning treatments, the decrease in TA content of peach fruits in the first year continued in the second year as well. In particular, in the summer pruning trees that made a higher rate reductions were identified (Table 3). Similar to the results of our research, Hossain and Mizutani [1] reported that in pruning treatments conducted on 9-year-old “Akatsuki” peach varieties budding on strong rootstock in 2001–2005 TA value decreased in trees with SP more than the decrease in those with WP. Decreased titratable acidity following pruning treatment probably was related to increased light penetration into the center of the trees. Furthermore, Hossain et al. [40] reported that SSC was higher and TA was lower in summer-pruned than in winter-pruned peach trees. Bayazit et al. [3] reported in a study conducted on peach and nectarine varieties that SP has no effect on SSC, pH, and TA values of fruits.

Carbohydrates are an essential source of reserve energy in temperate zone trees and other perennial plants. They can be mobilized for metabolism or translocated to other plant organs. The concentration and localization of carbohydrates, such as sugars and starches, within tissues are affected by many factors, such as temperature, moisture, light, pruning, and time of planting [20]. As can be seen in Table 4, it was found that, in pruning treatments conducted on two early season peach varieties, trees in control and WP treatment had highest values in terms of both starch and total extracted carbohydrate contents. The late SP had higher values in terms of similar carbohydrate components compared to those with early SP.

In SP treatments conducted on plenty of fruit varieties, many researchers reported that shoot reenlargement was observed in trees with early SP as to compensate decreasing leaf areas. Due to the shoot reenlargement on trees, decreases were observed in stored carbohydrates of trees. Lang [43] reported that SP conducted until harvest resulted in the decrease in storage reserves of trees to be used in following periods. Demirtaş et al. [44] conducted 5 different SP and WP treatments on “Hacıhaliloğlu” apricot trees and found that postharvest SP treatment has the highest increasing effect on average total sugar, reducing sugar, and starch contents. In sweet cherry, one year after SP, the level of carbohydrate in trunk was lower compared to unpruned trees [12]. Other studies have also shown that pruning results in quantitative changes in carbohydrate reserves. Pruning affects the concentration of reserves, by elimination of carbohydrate storage sites [18].

The results that we obtained about peach varieties related to carbohydrate contents are completely compatible with the findings of Stutte et al. [45], Danielle et al. [46], and İkinci [7] who studied on apple, cherry, apricot, and peach varieties.

## 5. Conclusion 

Pruning treatments performed on some temperate climate fruit varieties such as peach suggested that SP decreases tree length, improves fruit quality, thanks to better sunshine in tree crown, significantly increases marketable fruit percentage, and improves enhanced flower bud formation. As it is anticipated, peach agriculture will be improved in Şanlıurfa province in the near future. The focal point of this research was to control tree growth with pruning and obtain higher quality fruits.

The present study suggested that SP decreased shoot length, increased shoot diameter enlargement, decreased fruit yield, and increased fruit weight; and on condition that SP treatment is applied each year, SSC increased significantly and TA amount decreased. Preharvest summer pruning obviously promotes fruit maturation when compared to unpruned trees during the season of pruning. In addition, it was found that, in peach trees with SP, carbohydrate content significantly decreased compared to trees in control and only WP and this decrease was observed in preharvest SP treatment mostly.

We found that marketable fruit yields of trees with SP increased significantly, fruits got more colorful, and an observable increase was found in leaf area after 1-2 months of pruning treatments. Preharvest SP treatments on peach bring partial damages to fruits on trees. The effect of pruning treatments on tree and fruit quality can be observed within the next session. Our experience indicates that peach growers should consider SP as a standard cultural technique in the development of peach trees.

## Figures and Tables

**Table 1:** The influence of summer and winter pruning treatments on average shoot length and shoot diameter.

Pruning treatment	Early Red				Maycrest			
	Average shoot length (cm)		Shoot diameter (mm)		Average shoot length (cm)		Shoot diameter (mm)	
	2009	2010	2009	2010	2009	2010	2009	2010
Control	73.36 a^z^	67.12 a	12.1 a	11.4 b	70.88 a	72.17 a	12.2 ab	14.6 a
WP	68.64 a	61.35 b	10.6 b	12.3 a	75.08 ab	69.38 ab	13.5 a	12.4 b
SP-May	60.89 b	54.74 bc	9.2 bc	10.7 bc	66.38 b	52.72 b	10.7 b	11.4 bc
SP-June	55.15 bc	50.62 c	8.6 c	10.0 c	54.86 c	49.50 b	9.9 c	10.2 c
SP-July	47.84 c	40.08 d	9.2 bc	10.2 bc	45.71 c	44.43 b	9.4 c	9.8 c
SP-August	41.45 c	46.36 cd	8.7 c	9.7 c	49.27 c	45.48 b	9.6 c	9.4 c

^z^Mean separation within columns by Duncan's multiple range test at 5% level.

**Table 2:** The effects of summer and winter pruning time on TCSA, yield, and yield efficiency of “Early Red” and “Maycrest” peach cultivars.

Pruning treatment	TCSA (cm^2^)		Yield (kg tree^−1^)		Yield efficiency (kg cm^−2^ TCSA)	
	2009	2010	2009	2010	2009	2010
Early Red						
Control	156.78 a^z^	190.23 a	61.98 a	75.92 a	0.40 a	0.40 a
WP	140.11 b	172.79 b	43.62 bc	57.11 ab	0.31 b	0.33 b
SP-May	151.27 ab	178.17 ab	38.98 c	46.13 b	0.26 c	0.26 c
SP-June	137.71 b	160.96 c	52.48 ab	53.92 ab	0.38 ab	0.34 b
SP-July	146.91 ab	167.12 b	41.54 c	45.71 b	0.28 bc	0.27 c
SP-August	159.88 a	175.94 a	49.48 b	51.02 ab	0.31 b	0.29 c
Maycrest						
Control	130.7 ab	164.09 b	75.98 a	68.24 a	0.58 a	0.42 a
WP	146.22 a	175.29 a	65.24 ab	63.09 ab	0.45 ab	0.36 c
SP-May	126.67 b	152.06 c	53.12 b	52.23 c	0.42 b	0.34 c
SP-June	129.64 b	159.08 bc	53.94 b	62.68 ab	0.42 b	0.39 b
SP-July	119.29 c	142.37 d	50.4 b	57.79 b	0.42 b	0.41 ab
SP-August	134.73 ab	163.15 b	62.52 ab	59.93 b	0.46 ab	0.37 b

^z^Mean separation within columns by Duncan's multiple range test at 5% level.

**Table 3:** Average fruit weight (g), soluble solids (%), and titratable acidity (%) of peaches as influenced by summer and winter pruning.

Pruning treatment	Average fruit weight (g/fruit)		Soluble solids content (%)		Titratable acidity (%)	
	2009	2010	2009	2010	2009	2010
Early Red						
Control	78.74 d^z^	84.58 c	14.50	14.75	0.69 bc	0.62
WP	85.97 c	97.27 bc	14.65	15.25	0.65 c	0.63
SP-May	117.46 a	122.87 a	15.00	15.50	0.71 b	0.64
SP-June	99.46 bc	105.84 b	15.75	15.50	0.77 a	0.64
SP-July	110.05 b	100.47 b	15.00	14.75	0.73 ab	0.66
SP-August	92.79 c	115.89 ab	15.30	15.00	0.73 ab	0.64
Maycrest						
Control	87.30 b	99.79 b	14.60 ab	15.00	0.67 a	0.58 a
WP	100.28 b	117.92 ab	14.33 a	15.60	0.64 ab	0.59 a
SP-May	121.90 a	100.36 b	14.33 ab	14.90	0.59 bc	0.54 b
SP-June	108.17 ab	124.75 a	14.67 b	15.25	0.62 b	0.53 b
SP-July	103.29 ab	110.58 b	15.07 a	15.70	0.57 c	0.51 b
SP-August	106.36 ab	105.05 b	15.53 a	15.20	0.56 c	0.51 b

^z^Mean separation within columns by Duncan's multiple range test at 5% level.

**Table 4:** The influence of pruning treatments on concentration of starch and total carbohydrate content of shoot of “Early Red” and “Maycrest” peach cultivars.

Pruning treatment	Early Red				Maycrest			
	Starch (%)		Total extracted CHO (%)		Starch (%)		Total extracted CHO (%)	
	2009	2010	2009	2010	2009	2010	2009	2010
Control	5.50 a^z^	6.80 a	8.46 a	9.96 a	5.83 a	6.78 a	8.62 a	9.81 a
WP	5.11 ab	6.50 ab	7.94 ab	9.63 ab	5.55 a	6.41 ab	8.57 a	9.30 ab
SP-May	4.50 c	5.24 c	7.03 c	7.70 c	4.22 c	4.85 d	6.36 d	7.20 d
SP-June	4.12 d	5.17 c	6.57 c	7.45 c	4.70 b	4.98 cd	7.06 c	7.42 c
SP-July	4.63 c	5.94 b	7.28 c	8.67 b	4.80 b	5.35 c	7.39 bc	8.03 c
SP-August	4.72 b	6.29 b	7.44 b	9.10 b	5.04 ab	6.02 b	7.67 b	8.85 b

^z^Mean separation within columns by Duncan's multiple range test at 1% level.
